# Impact of the COVID-19 pandemic on sexually transmitted infection testing and diagnosis in Lebanon: A retrospective chart review

**DOI:** 10.1016/j.heliyon.2024.e39191

**Published:** 2024-10-10

**Authors:** Nadine Sunji, Peter Boufadel, Iman Fakih, Jana Haidar Ahmad, Mathieu Choufani, Nabih Habib, Jean-Paul Rizk, Ryan Yammine, Sara Abu Zaki, Ayman Assi, Laith J. Abu-Raddad, Sasha Fahme, Ghina R. Mumtaz

**Affiliations:** a Faculty of Medicine, American University of Beirut, Beirut, Lebanon; b Department of Epidemiology and Population Health, American University of Beirut, Beirut, Lebanon; c Marsa Sexual Health Center, Beirut, Lebanon; d Faculty of Medicine, Saint Joseph University of Beirut, Beirut, Lebanon; e Infectious Disease Epidemiology Group, Weill Cornell Medicine-Qatar, Cornell University, Qatar Foundation – Education City, Doha, Qatar; f World Health Organization Collaborating Centre for Disease Epidemiology Analytics on HIV/AIDS, Sexually Transmitted Infections, and Viral Hepatitis, Weill Cornell Medicine–Qatar, Cornell University, Qatar Foundation–Education City, Doha, Qatar; g Department of Population Health Sciences, Weill Cornell Medicine, Cornell University, New York, NY, USA; h Department of Public Health, College of Health Sciences, QU Health, Qatar University, Doha, Qatar; i College of Health and Life Sciences, Hamad bin Khalifa University, Doha, Qatar; j Center for Infectious Diseases Research, Faculty of Medicine, American University of Beirut, Beirut, Lebanon

**Keywords:** Sexually transmitted infections, COVID-19, Sexual behavior, Lebanon

## Abstract

**Background:**

Social distancing restrictions during the COVID-19 pandemic caused disruptions to sexual health services (SHS) worldwide. During the first year of the pandemic, Lebanon implemented multiple lockdowns during which SHS endured repetitive closures. We explore the impact of the pandemic on SHS delivery and the diagnosis rate of sexually transmitted infections (STIs) among attendees of a large sexual health clinic in Beirut, Lebanon.

**Methods:**

This was a retrospective analysis of the clinic's database, including data on voluntary counseling and testing (VCT) for HIV, syphilis, hepatitis B virus (HBV), and hepatitis C virus (HCV). We compared the number and types of services provided, and the number and rate of positive VCT diagnoses pre- (Mar 2019–Feb 2020) and post- (Mar 2020–Feb 2021) COVID-19 onset.

**Results:**

Men who have sex with men (MSM) comprised 35 % and 40 % of attendees pre- and post- COVID-19 onset, respectively. Post-COVID-19 onset, a total of 1350 VCT services and 406 medical consultations were provided, an overall 45 % decrease compared with pre-COVID-19 onset. The prevalence pre-COVID-19 onset of HIV, syphilis, HBV, and HCV was 0.8 %, 0.3 %, 0.2 %, and 0.1 %, respectively, and post-COVID-19 onset 1.2 %, 0.7 %, 0.3 %, and 0.3 %, respectively. Post-COVID-19 onset, 1.7 % of patients tested positive for any STI compared with 1.1 % pre-COVID-19 onset (OR: 1.5, 95%CI: 0.8–2.7). Close to 90 % of all positive diagnoses were among MSM. The prevalence of HIV, syphilis, HBV, and HCV among MSM in the total sample was 2.1 %, 1.2 %, 0.4 %, and 0.3 %, respectively.

**Conclusion:**

COVID-19 related closures led to substantial reduction in SHS accessibility among clinic attendees. STI positivity rates increased post-COVID-19 onset, although this increase was not statistically significant. Findings suggest that sexual risk behavior was taking place during the pandemic despite the lockdowns and highlight the need to minimize disruptions in provision of SHS during similar crises, particularly to key populations.

## 1 Background

Sexual health services (SHS) globally were dramatically reduced during the COVID-19 pandemic, limiting access to sexually transmitted infection (STI) screening, prevention, and treatment [[1], [2], [3], [4], [5], [6], [7], [8], [9], [10], [11], [12], [13]]. Although these disruptions were widespread, they were especially pronounced in low- and middle-income countries (LMICs), where HIV testing and antiretroviral therapy (ART) initiation faced the greatest challenges [[7], [8], [9], [10], [11], [12], [13]]. A recent systematic review found a greater decline in HIV testing rates among LMICs as compared to high-income countries, potentially due to more stringent lockdown measures and/or the added strain on already-fragile health systems confronted with compounding crises [8].

Despite disruptions in SHS, a simultaneous decline in sexual activity and sexual risk behavior during the first year of the pandemic [[14], [15], [16]], including among key populations such as men who have sex with men (MSM) [[17], [18], [19], [20]], may have mitigated the otherwise heightened risk of STI transmission. Several mechanisms likely contributed to these behavioral shifts, including the reduced opportunities for casual sexual encounters due to the closure of public venues such as bars and other social spaces, limitations on face-to-face interactions and social mingling, and the fear of contracting COVID-19 which may have led many individuals to voluntarily reduce their sexual activity or avoid new sexual partners [21,22]. The unavailability of condoms and HIV pre-exposure prophylaxis due to supply chain disruptions and service access challenges may have also influenced sexual behavior patterns. Furthermore, changes in social and psychological conditions during the pandemic—such as heightened anxiety, economic stress, and isolation—may have contributed to reductions in sexual activity [14,23]. These factors collectively reduce sexual risk behaviors, thereby potentially curbing STI transmission during this period.

Indeed, there was a reduction in the number of new STI diagnoses reported in several high-income countries in the early months of the pandemic [[24], [25], [26], [27], [28], [29]], though data from the Global South are scarce. One study in Mexico reported an increase in the number of notified urogenital gonorrhea cases during the pandemic [30], while a decrease in the reporting of HIV diagnoses was documented in Peru [31]. There are especially limited data characterizing the impact of the COVID-19 pandemic on STI prevalence and incidence in the Middle East and North Africa (MENA) region, where STI epidemiology has long been understudied and remains generally poorly understood [32].

There is a dearth of published research on sexual health in the MENA region. Available studies indicate an overall lower prevalence of STIs compared with other world regions [[33], [34], [35], [36]], but with a disproportionate burden among MSM and female sex workers [34,[37], [38], [39], [40], [41]]. Furthermore, MENA is one of three world regions with an increasing incidence of HIV infection and consistently ranks lowest on key HIV response indicators set by UNAIDS [42]. Between 2010 and 2022, the region witnessed a striking 61 % upsurge in new HIV infections, ranking it as the world's most substantial regional increase in infection rates [42]. In the two decades prior to the COVID-19 pandemic, protracted conflict and forced displacement across the region may have increased vulnerability to poor sexual health outcomes including HIV and other STIs [[43], [44], [45], [46]], though a paucity of data limits our understanding of how such crises impact sexual health.

Lebanon, a lower-middle income country in the MENA region, has recently endured several compounding crises including ongoing political instability, an unprecedented economic collapse, and the August 2020 explosion of the Beirut Port. Health systems were duly destabilized by mass emigration of physicians and other healthcare professionals, sustained shortages of fuel and basic medical supplies including medications, and an overall inadequacy of resources due to the devaluation of the local currency [47,48]. The pandemic and its associated restrictions further destabilized the country's fragmented health system and impacted SHS delivery [45,49]. After Lebanon confirmed its first COVID-19 case on February 21, 2020 [50], aggressive nationwide measures including stringent lockdowns and border closures were implemented to curb the spread of the virus. Health services considered “non-essential” were halted, leading to the closure of sexual health clinics [51]. However, the precise impact of these crises on SHS delivery and STI transmission has not been studied. While data elsewhere indicated a decline in sexual activity [[24], [25], [26], [27], [28], [29]], a preliminary assessment of the impact of COVID-19 on sexual health among MSM clinic attendees in Beirut during the early months of the pandemic suggested ongoing risky sexual activity despite the enforcement of lockdown measures [49].

The purpose of this study was to: 1) quantify the reduction in SHS rendered during the first year of the COVID-19 pandemic at one of the largest sexual health clinics in Lebanon, 2) estimate the prevalence of HIV, syphilis, hepatitis B virus (HBV), and hepatitis C virus (HCV), pre- and post-COVID-19 onset, among attendees of this clinic, and 3) assess the impact of the pandemic on the diagnosis rate of these infections among these attendees.

## 2 Materials and methods

### 2.1 Study design

We conducted a retrospective analysis of the de-identified clinical database of the Marsa sexual health center in Beirut between March 1, 2019 and February 28, 2021. We followed STROBE guidelines for the reporting of cross-sectional studies [52]. Marsa has been operating as a non-profit organization since 2011 and provides comprehensive sexual and reproductive health services to all individuals, irrespective of gender and sexual orientation [53,54]. Given their services are inclusive and void of stigma and discrimination against sexual practices, the clinic is highly accessible for MSM who comprise a sizable fraction of their patient population. Services comprise voluntary counseling and testing (VCT) and physician evaluation (medical consultation). VCT includes rapid testing for HIV, syphilis, HBV, and HCV. Gonorrhea, chlamydia, and trichomoniasis are not tested at this clinic due to the high costs of the testing kits, but are treated by attending physicians based on symptoms. Most of the center's services are offered free of charge or at a subsidized price. During the COVID-19 lockdowns, the center functioned at reduced capacity, providing occasional telehealth consultations for symptomatic patients and accommodating certain emergencies (e.g., urgent need for testing).

We compared medical records in the year prior to COVID-19 pandemic onset in Lebanon (March 1, 2019 to February 29, 2020) with those in the first year of the COVID-19 pandemic (March 1, 2020 to February 28, 2021). Nationwide lockdowns in Lebanon were imposed from March 2020 to May 2020 and January 2021 to February 2021.

### 2.2 Analysis

We extracted the following information from the clinic's database: Type of service provided (VCT vs. medical consultation), rapid test results, and patient socio-demographic characteristics including age, sex assigned at birth, gender, highest level of education, occupation, and sexual practice. Sexual practice was classified into four categories: 1) Men who have sex only with women, 2) men who have sex with men (including men who have sex with men and women), 3) women who have sex only with men, and 4) women who have sex with women (including women who have sex with women and men).

Analyses were conducted at the service level and patient level. To assess the impact of the pandemic on service provision and access, service-level analyses were conducted. All records indicating a VCT or medical consultation during the study periods were included. No records were excluded. The number of services (VCT and medical consultations) provided at the clinic was compared pre- and post-COVID-19 onset, both monthly and aggregated by study period. We also compared the aggregate number of services post-COVID-19 onset in July–December 2020 when the lockdown was lifted with respective calendar months pre-COVID-19 onset.

To describe the study population and assess the impact of the pandemic on STI positivity rates, patient-level analyses were conducted. All clinic attendees receiving a VCT service during the study periods were included in the analyses. For the purposes of this study, VCT services were defined as rapid testing for HIV, syphilis, hepatitis B virus (HBV), and hepatitis C virus (HCV). No records were excluded. Repeat patients were included only once per study period. Distribution of socio-demographic characteristics and sexual practices were reported pre- and post-COVID-19 onset for non-missing data using frequencies and proportions. Percent missing data was reported for variables if it was greater than 10 % - only the occupation variable met this criterion. The Chi-square test was used to assess differences in distributions between the pre- and post-COVID-19 onset periods. P-values were reported.

Positivity rates were calculated for STIs HIV, syphilis, and HBV – individually and combined) and HCV using all rapid testing records per patient per study period. Positivity rates were not calculated for any infections that were diagnosed syndromically, including gonorrhea, chlamydia, and trichomoniasis, as no testing was not conducted for these infections. Positivity rates were compared pre- and post-COVID-19 onset using univariate logistic regression. Generalized estimating equations with autoregressive structures were employed to account for individuals that appear in both the pre- and post-COVID-19 onset periods. Odds ratios (ORs) and 95 % confidence intervals (CIs) were reported.

All analyses were performed using R 4.3.0 [55].

## 3 Results

### 3.1 Number of services

**Fig. 1:**
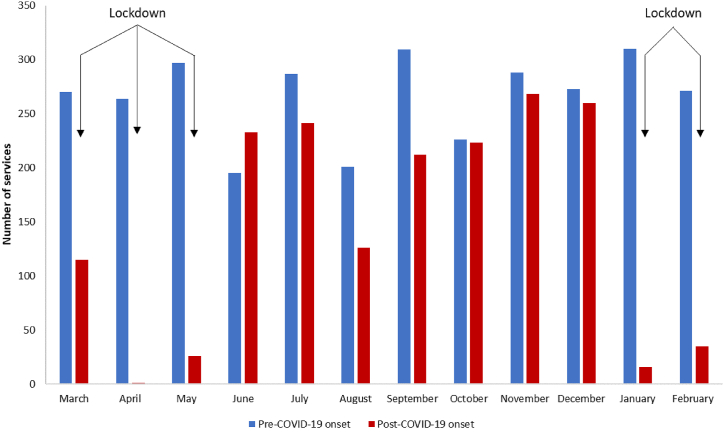
Number of services provided by Marsa clinic by calendar month, stratified by pre- and post-COVID-19 onset. Note: The pre-COVID-19 period extends from March 1, 2019 to February 29, 2020, and the post-COVID-19 period extends from March 1, 2020 to February 28, 2021.

A total of 1756 services were provided in the post-COVID-19 onset period compared with 3191 services pre-COVID-19 onset, indicating a 45 % decrease (Table 1, Fig. 1). Each month (except June 2020) during the pandemic saw fewer services delivered than in the corresponding months prior to COVID-19 (Fig. 1), with the most significant declines observed during the national lockdowns in 2020 (March–May) and early 2021 (January–February) (Fig. 1). While the majority of services post-COVID-19 onset (75.7 %; n = 1330) were offered during the 6 months outside of lockdown, there was still a 16 % reduction in services in these months compared to the same calendar months before COVID-19 onset (Table 1, Fig. 1). A noticeable increase in the number of services was observed in June 2020 when the lockdown was lifted, surpassing the service count for the same month pre-COVID-19 onset (233 vs. 195, respectively) (Fig. 1). The distribution of services by type remained consistent in both study periods, with approximately 80 % consisting of VCT and 20 % being medical consultations (Table 1).

**Table 1:** Number and type of services provided at Marsa sexual clinic during the entire study period and during months outside of lockdown, stratified by pre- and post-COVID-19 onset.

	**Pre-COVID-19 onset**	**Post-COVID-19 onset**
**Entire study period**	Mar 2019–Feb 2020	Mar 2020–Feb 2021
Number of services	3191	1756
VCT, n (%)	2532 (79.3)	1350 (76.9)
Medical consultations, n (%)	659 (20.7)	406 (23.1)
**Months outside lockdown**^a^	July–Dec 2019	July–Dec 2020
Number of services	1584	1330
VCT, n (%)	1228 (77.5)	1035 (77.8)
Medical consultations, n (%)	356 (22.5)	295 (22.2)

COVID-19: coronavirus disease 2019; VCT: voluntary counseling and testing.

a June 2020 was considered a transition month during which the lockdown was gradually lifted and hence was not included as part of the months outside of lockdown.

### 3.2 Study population characteristics

During the study period, the majority of clinic attendees were male by birth assignment (64.7 %), Lebanese (78.7 %), and university-educated (89.1 %), with a median age of 27 years (Interquartile range: 23–32 years). Almost all attendees (98.3 %) identified as cis-gender, and 35.2 % were MSM (Table 2).

**Table 2:** Sociodemographic characteristics and sexual practices of Marsa clinic attendees, stratified by pre- and post-COVID-19 onset.

	Overall^a^ (Mar 2019–Feb 2021)	Pre-COVID-19 onset (Mar 2019–Feb 2020)	Post-COVID-19 onset (Mar 2020–Feb 2021)	p-value^b^
	N = 3106	N = 2180	N = 1191	
	n (%)	n (%)	n (%)	
**Age (years)**				0.254
<20	122 (3.9)	90 (4.1)	37 (3.1)	
20-24	875 (28.2)	601 (27.6)	347 (29.2)	
25-29	1071 (34.5)	760 (34.9)	395 (33.2)	
30-34	520 (16.7)	360 (16.5)	224 (18.8)	
35-39	276 (8.9)	198 (9.1)	98 (8.2)	
>=40	241 (7.8)	171 (7.8)	89 (7.5)	
**Assigned sex at birth**				0.873
Male	2009 (64.7)	1435 (65.8)	788 (66.2)	
Female	1097 (35.3)	745 (34.2)	403 (33.8)	
**Nationality**				<0.001
Lebanese	2436 (78.7)	1726 (79.4)	932 (78.5)	
Syrian	334 (10.8)	196 (9.0)	161 (13.6)	
Palestinian	72 (2.3)	56 (2.6)	22 (1.9)	
Other Arab	51 (1.6)	40 (1.8)	15 (1.3)	
Non-Arab	204 (6.6)	156 (7.2)	58 (4.9)	
**Highest degree**				<0.001
University	2565 (89.1)	1840 (91.8)	962 (85.1)	
Technical	55 (1.9)	26 (1.3)	31 (2.7)	
Secondary	93 (3.2)	54 (2.7)	46 (4.1)	
Primary	101 (3.5)	39 (1.9)	70 (6.2)	
Other	65 (2.3)	46 (2.3)	22 (1.9)	
**Occupation**^c^				<0.001
Employed	889 (46.7)	597 (46.4)	379 (48.0)	
Student	683 (35.9)	493 (38.3)	246 (31.2)	
Jobless/Between jobs	282 (14.8)	157 (12.2)	149 (18.9)	
Other	65 (2.3)	46 (2.3)	22 (1.9)	
**Gender**				0.033
Cis-male	1960 (64.0)	1413 (65.4)	757 (65.0)	
Cis-female	1050 (34.3)	723 (33.4)	378 (32.4)	
Transgender	37 (1.2)	18 (0.8)	21 (1.8)	
Other	65 (2.3)	46 (2.3)	22 (1.9)	
**Sexual practice**				0.043
MSW	926 (30.4)	673 (31.2)	319 (27.6)	
MSM	1074 (35.2)	760 (35.2)	461 (39.8)	
WSM	916 (30.0)	634 (29.4)	326 (28.2)	
WSW	134 (4.4)	90 (4.2)	51 (4.4)	

COVID-19: coronavirus disease of 2019; MSM; men who have sex with men; MSW: men who have sex with women; WSM: women who have sex with men; WSM: women who have sex with men; WSW: women who have sex with women.

a Patients who appeared in both the pre- and post-COVID-19 onset periods were counted once in the overall sample.

b p-value was generated using Chi-square tests comparing the pre- and post-COVID-19 onset distributions.

c For the occupation variable, 38.5 % of the sample had missing data. All other variables have less than 7 % missing data.

There were no statistically significant differences in age and sex assigned at birth between the pre- and post-COVID-19 onset periods (Table 2). Post-COVID onset, there was a decrease in the proportion of non-Arab attendees (4.9 % vs. 7.2 %) and an increase in Syrian attendees (13.6 % vs. 9.0 %) (p < 0.001). The post-COVID onset period also saw a higher percentage of jobless attendees (18.9 % vs. 12.2 %) and a lower percentage of students (31.2 % vs. 38.3 %) (p < 0.001). Attendees had generally lower educational attainment in the post-COVID onset period (p < 0.001), with fewer having a university degree (85.1 % vs. 91.8 %) and more having only completed primary school (6.2 % vs. 1.9 %).

After the onset of COVID-19, the proportion of attendees who identify as transgender increased from 0.8 % to 1.8 % (p = 0.033) and there was an increase in the proportion of attendees who were MSM (39.8 % vs. 35.2 %; p = 0.043).

### 3.3 STI and HCV testing and positivity rates

In the post-COVID-19 period, 1191 clinic attendees underwent STI testing, compared with 2180 in the pre-COVID-19 period, representing a 45.4 % decrease (Table 3). This reduction in testing was observed across all types of STIs. The prevalence pre-COVID-19 onset of HIV, syphilis, HBV, and HCV was 0.8 %, 0.3 %, 0.2 %, an 0.1 %, respectively; while the prevalence of these infections post-COVID-19 onset was 1.2 %, 0.7 %, 0.3 %, and 0.3 %, respectively.

**Table 3:** Comparison of STI and HCV testing numbers and positivity rates pre- and post-COVID-19 onset.

	**Pre-COVID-19 onset (Mar 2019 – Feb 2020)**		**Post-COVID-19 onset (Mar 2020 – Feb 2021)**		Positivity OR (95 % CI)
	Tested, n	Positive, n (%)	Tested, n	Positive, n (%)	
Any STI	2180	25 (1.1)	1191	20 (1.7)	1.5 (0.8–2.7)
HIV	2166	17 (0.8)	1186	14 (1.2)	1.5 (0.7–3.1)
Syphilis	1749	5 (0.3)	1025	7 (0.7)	2.4 (0.8–7.6)
HBV	1694	4 (0.2)	988	3 (0.3)	1.3 (0.3–5.8)
HCV	1718	2 (0.1)	376	1 (0.3)	2.3 (0.2–25.3)

CI: confidence interval; COVID-19: coronavirus disease of 2019; HBV: hepatitis B virus; HCV: hepatitis C virus; HIV: human immunodeficiency virus; OR: odds ratio; STI: sexually transmitted infection.

The overall positivity rate for any of the tested STIs (HIV, syphilis, and HBV) increased from 1.1 % to 1.7 %, but this increase was not statistically significant (OR = 1.5, 95 % CI: 0.8–2.7). A similar trend was observed for all individual STIs. The odds of testing positive for HIV, syphilis, and HBV in the post-COVID-19 period were, respectively, 1.5 (95 % CI: 0.7–3.1), 2.4 (95 % CI: 0.8–7.6), and 1.3 (95 % CI: 0.3–5.8) times higher when compared with the pre-COVID-19 period (Table 3). Out of the 45 cases that tested positive for any STI in the entire study period, 44 (97.8 %) were male by birth assignment, and 39 (86.7 %) were MSM. The proportion of MSM among those who tested positive for any STI was unchanged between the pre- and post-COVID-19 periods. The prevalence of HIV, syphilis, and HBV among MSM during the entire study period was 2.1 %, 1.2 %, and 0.4 %, respectively.

Similarly, there was a large decline in the number of HCV tests post-COVID-19 onset (376 vs. 1718; 78 % reduction) and a noticeable increase in HCV positivity rates, though this increase was not statistically significant (OR: 2.3, 95 % CI: 0.2–25.3) (Table 3).

## 4 Discussion

This study indicates a substantial impact of COVID-19-related closures on SHS accessibility for attendees at this sexual health clinic in Beirut, Lebanon. There was large reduction in both medical consultations and testing services during the first year of the pandemic, in the setting of two periods of national lockdowns, marking a deviation from the clinic's steady growth in service provision over the past decade. This substantial decline in the number of services can be attributed to the clinic's focus on urgent and symptomatic cases during strict closures, with routine screening and consultations available only during non-lockdown months. A similar reduction in testing was reported by Maatouk (2020) during the early months of the pandemic at a private dermatology clinic in Beirut [49].

The rebound in service utilization during the month when the lockdown was lifted (June 2020) suggests that sexual activity and demand for SHS persisted during the lockdown period. Consistently, Maatouk (2020) reported an increase in demand for preexposure prophylaxis for HIV during the lockdown, confirming ongoing sexual risk-taking [49]. This underscores the notion that while the pandemic disrupted service delivery, it did not supress the need for sexual health support. A similar rebound effect was observed in other settings that faced SHS interruptions during lockdowns and other pandemic-related restrictions [2,17,29,56]; while settings with uninterrupted services, like a sexual health clinic in Victoria, Australia, did not experience delayed health-seeking behavior [57].

Despite this evident persistence in SHS needs, demand for clinic services during the months outside of lockdown still remained lower than pre-COVID-19 levels (16 % decrease), which could be attributed to a change in health-seeking behavior, possibly due to concerns about contracting COVID-19 within healthcare settings [58]. While this reduction could also reflect a decrease in sexual risk behavior during the pandemic, the positivity rate in this time period suggests otherwise.

There was a tendency for higher STI positivity rates for all STIs, especially syphilis, in the post-COVID-19 onset period. This may reflect an actual increase in STI transmission, potentially resulting from reduced access to prevention and treatment services and/or an increase in sexual activity as a means of coping with the adverse mental health outcomes associated with the pandemic and other simultaneous crises in the country [59]. The higher positivity rate could also reflect engagement in sexual risk-taking, such as sex work, as the concurrence of the pandemic and its economic disruptions with an enduring economic crisis may have exacerbated financial insecurity. Due to devaluation of the local currency, prices of basic goods and services soared while income did not change to match. Rates of transactional and survival sex have been shown to increase in vulnerable populations during times of economic instability [11,60,61]. Studies have also shown that despite pandemic-related restrictions, high-risk sexual behaviors persisted among MSM in Kenya, Brazil, Portugal, and the UK [19,62,63].

Perhaps more likely, the apparent increase in STI positivity rates during the pandemic could be due to a shift in the clinic's patient population, with mostly individuals at higher risk of STI seeking SHS. The data indicate that post-COVID-19 onset, a more vulnerable patient cohort was seeking testing – characterized by lower education levels, increased unemployment rates, a higher proportion of refugees, and a higher proportion of MSM and transgender individuals. The baseline STI positivity rate of this vulnerable population may have remained unchanged and only became overrepresented in the clinic's attendees post-COVID-19 onset. The substantial reduction in asymptomatic screening during lockdowns, when services were limited to symptomatic and urgent cases, also likely contributed to the observed STI positivity rate post-COVID-19 onset. Rising symptomatic STI rates, in contrast with lower rates of early diagnosed asymptomatic cases, were observed in Australia and the US during the pandemic [26,27,57,64].

The pandemic and related-closures disproportionately affected the most vulnerable populations [65]. MSM comprise about 40 % of the patient population of this sexual health clinic; and the vast majority of STI cases before and after COVID-19 onset were identified among MSM. MSM are a highly stigmatized population in Lebanon with limited access to HIV and STI prevention and treatment services. Marsa is one of only a handful organizations providing SHS to MSM in Lebanon, an estimated population of approximately 16,500 men [66]. This is likely an underestimate given the hidden nature of the population and the lack of recent and methodologically robust risk group size estimation studies. The closure of sexual health clinics during crises such as the COVID-19 pandemic further compounds the vulnerability of MSM and other marginalized groups to STIs. This is particularly concerning given the documented concentrated HIV epidemics among MSM in Lebanon [67], and findings of mathematical modeling studies linking pandemic-related disruptions in HIV and STI care with increased incidence of these infections, including in LMIC [[68], [69], [70], [71]].

Irrespective of the impact of COVID-19, the prevalence of HIV, syphilis, and HBV among MSM in this sample at 2.1 %, 1.2 %, and 0.4 %, respectively, was overall lower than what was previously reported in the very few studies conducted among MSM in Lebanon [43,54,72,73]. In the period 2015–2018, the prevalence of HIV, syphilis, and HBV among MSM attending Marsa sexual health center was 5.6 %, 3.0 %, and 0.5 % respectively [54]. A similar HIV prevalence of 5.95 % was found among MSM attending another sexual health clinic during the period 2014–2018 [72]. Integrated bio-behavioural surveillance surveys using probability-based sampling techniques conducted in 2014–2015 reported an HIV prevalence of 12.3 % among MSM [73] and 2.7 % among MSM refugees in Beirut [43]. More recent estimates are not available. The prevalence of HIV and syphilis in this sample of MSM was also lower than estimates for MSM in the MENA region [40,67,74] and globally [74,75].

This study has limitations. Information was restricted to variables available in the clinic's medical records. Additionally, the relatively small number of positive STI cases limited any further statistical analyses, such as multivariable regression analyses, that may have improved our interpretation of STI trends associated with COVID-19. It was also not possible to conclude whether the pandemic did not actually impact STI positivity rate, given statistical non-significance, or if the effect was undetected due to the insufficient sample size. The small number of cases also prevents us from generalizing the sociodemographic characteristics of positive cases. While Marsa is one of the largest sexual health clinics in the country and a substantial fraction of the patient population are MSM, findings cannot be generalized to the wider MSM community in Lebanon; sexual behavior and STI trends may be different among MSM with different health-seeking behaviors. Gonorrhea, chlamydia, and trichomoniasis, three important STIs with typically a significant incidence among such facility-based populations, could not be included in the study because they are diagnosed and treated solely based on symptoms due to the high costs of the testing kits.

Another limitation of the study is the inability to adjust for the confounding effects of Lebanon's economic and political crises, as well as the August 4, 2020 Beirut Port explosion. Although the political instability and financial collapse began prior to the COVID-19 pandemic (around October 2019), the multiple crises may have acted as confounders by increasing financial vulnerability and serving as psychological stressors. These factors may have impacted sexual risk-taking and health-seeking behaviors, making it difficult to isolate the specific impact of the COVID-19 pandemic on STI positivity. Moreover, the crises could have contributed to the reduction in services even during the non-lockdown months and may have led to changes in the sociodemographic composition of the patient population, which became more vulnerable in the post-COVID-19 onset period. The worsening economic conditions introduced new barriers to healthcare access [48], as evidenced by the sharp rise in poverty (from 10.0 % of the population in 2018 to 55.3 % in 2020 [76]) and the escalation of unemployment rates (from 11.4 % in 2018–2019 to 29.6 % in 2022 [77]).

In conclusion, the study documents the substantial disruptions in SHS provision caused by the COVID-19 pandemic and their potential impact on STI transmission in Lebanon, particularly among vulnerable groups such as MSM. These findings have important implications for public health policy, especially in preparing for future pandemics or similar crises. SHS must be recognized as essential, with policies ensuring their continued accessibility during public health and other crises [13]. This is critical in the MENA region, which consistently ranks lowest on all indicators of the HIV treatment cascade [42] and cannot afford further set backs. Remote services like telemedicine consultations and self-testing should be integrated into routine healthcare practices, even outside emergencies. This would also help address the stigma associated with seeking care for sexual needs, especially in conservative settings. Investment should be made to strengthening the resilience of health systems to withstand future crises, including by training healthcare workers to provide remote sexual health services and securing supply chains for essential sexual health products [13]. Ensuring that SHS are part of broader emergency preparedness plans will reduce the negative impact of future crises on sexual health outcomes.

Additionally, lessons learned from changes in sexual behavior during the pandemic must be integrated into future public health policies. Studies have shown that lockdowns and social distancing measures led to shifts in sexual behavior, with some individuals reducing sexual contacts while others turned to alternative means of sexual expression, such as the increased purchase of sex toys and engagement with virtual sexual platforms [23,78]. These shifts in behavior demonstrate the adaptability of sexual practices in response to crisis conditions and emphasize the need for SHS to be flexible in addressing changing demands. Health systems should be prepared to meet these evolving needs, both during and beyond emergencies. Given that SHS in Lebanon are predominantly delivered by non-governmental organizations (NGOs), national public health strategies should prioritize strengthening financial and operational support for these NGOs to effectively implement responsive measures. These measures include expanding access to sexual health products, such as condoms and home-use STI testing kits, and facilitating the remote delivery of SHS to ensure that vulnerable populations can continue accessing care even when traditional healthcare services are disrupted.

Further research is warranted to investigate the longer-term impacts of interruptions in SHS caused by the pandemic and the other humanitarian, political, and economic crises in Lebanon and the broader MENA region. This would allow for a more nuanced understanding of the potentially evolving landscape of STI transmission in these challenging contexts. Qualitative data from both clinic attendees and healthcare providers would provide valuable insights into the specific barriers to accessing SHS during the pandemic and in non-crisis periods. Such data could help to better understand the challenges related to service disruptions, such as stigma, perceptions of risk, logistical difficulties, or financial constraints, and would inform strategies to improve SHS accessibility and delivery in future public health emergencies as well as in routine healthcare settings. Finally, enhanced surveillance of HIV and other STIs, particularly among key populations and individuals with increased vulnerability, is critically needed in Lebanon given the scarcity of available data.

## CRediT authorship contribution statement

**Nadine Sunji:** Writing – original draft. **Peter Boufadel:** Writing – original draft. **Iman Fakih:** Writing – original draft, Formal analysis. **Jana Haidar Ahmad:** Writing – review & editing, Resources, Formal analysis. **Mathieu Choufani:** Writing – review & editing, Resources, Formal analysis. **Nabih Habib:** Writing – review & editing, Resources, Formal analysis. **Jean-Paul Rizk:** Writing – review & editing, Resources, Formal analysis. **Ryan Yammine:** Writing – review & editing, Resources, Formal analysis. **Sara Abu Zaki:** Writing – review & editing, Methodology, Data curation. **Ayman Assi:** Writing – review & editing, Methodology, Data curation. **Laith J. Abu-Raddad:** Writing – review & editing, Methodology, Funding acquisition, Conceptualization. **Sasha Fahme:** Writing – original draft. **Ghina R. Mumtaz:** Writing – review & editing, Supervision, Methodology, Formal analysis, Funding acquisition, Conceptualization.

## 5 Ethics statement

Ethical approval to conduct this study was obtained from the Institutional Review Board of the American University of Beirut (ID: SBS-2020-0528, January 15, 2021).

## 6 Data and code availability

The data that has been used is confidential.

## 7 AI statement

During the preparation of this work the authors used ChatGPT in order to improve language. After using this tool, the authors reviewed and edited the content as needed and take full responsibility for the content of the publication.

## Declaration of competing interest

The authors declare that they have no known competing financial interests or personal relationships that could have appeared to influence the work reported in this paper.
